# Experimental review of PEI electrodeposition onto copper substrates for insulation of complex geometries†

**DOI:** 10.1039/d1ra05448a

**Published:** 2021-10-26

**Authors:** J.-C. Zirignon, A. J. Capezza, X. Xiao, R. L. Andersson, M. Forslund, P. Dinér, R. T. Olsson

**Affiliations:** KTH Royal Institute of Technology, CBH, Fibre and Polymer Technology Department Teknikringen 58 SE-100 44 Stockholm Sweden ajcv@kth.se rols@kth.se; Materials Technology Department, YTME, Scania CV AB Södertälje Sweden; KTH Royal Institute of Technology, CBH, Department of Chemistry Teknikringen 30 SE-100 44 Stockholm Sweden

## Abstract

Polyetherimide (PEI) was used for coating copper substrates *via* electrophoretic deposition (EPD) for electrical insulation. Different substrate preparation and electrical field application techniques were compared, demonstrating that the use of a pulsed voltage of 20 V allowed for the best formation of insulating coatings in the 2–6 μm thickness range. The results indicate that pulsed EPD is the best technique to effectively coat conductive substrates with superior surface finish coatings that could pass a dielectric withstand test at 10 kV mm^−1^, which is of importance within the EV automotive industry.

## Abstract 2

Electrophoretic deposition relying on electrodeposition of charged polymers *via* modulated electrical fields is reported. Superior surface finishes that could pass a dielectric withstand test at 10 kV mm^−1^ were obtained for pulsed potentials at 20 V.

## Graphical abstract

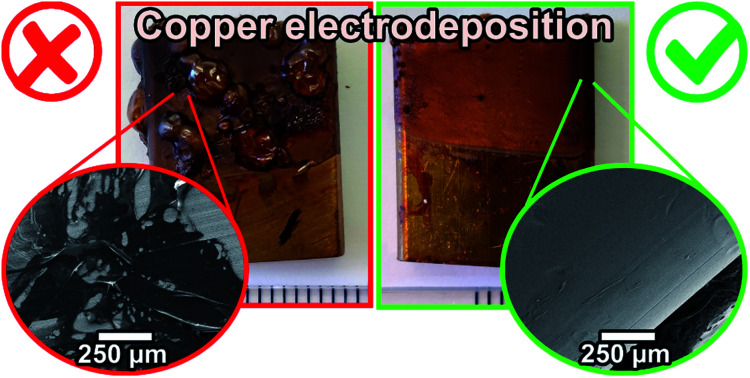

Electrophoretic deposition (EPD) is a useful and scalable technique for the production of electrically insulating coatings on conductive substrates having irregular shapes, while at the same time allowing a precise control of the coating thickness.^1,2^ EPD relies on the preparation of an aqueous colloidal suspensions based on charged polymers that are deposited as particles onto an electrically conductive substrate by an induced electric field.^3^ In contrast to other electrical field induced deposition methods such as electroplating, EPD does not require the suspension to have high electrical conductivity, allowing for small power losses due to the total current being used for the coating formation.^4^ Advantages also include that more complex shapes/parts can be coated due to the emulsion being able to reach into difficult geometries, consequently covering areas in hard to reach segments that otherwise would remain uncoated in a dip-coating procedure.^1–4^

To promote high electrophoretic movement of the polymer particles and homogenous formation of the polymer coatings, both high polymer charge and high polymer colloidal suspension concentration are required in the coating system.^5–7^ These factors are considered the most important in the selection of polymer for the EPD systems.^4^ The control over the coating quality has shown, however, to rely on several more parameters, including deposition time, electric field strength, suspension viscosity, applied voltage, *etc.*^7^ To enhance the polymer charge and solubility, functional groups have been added to the polymer structure and/or a mixture of solvents have been utilized, respectively.^8,9^ Furthermore, the thermal stability of the selected polymers needs to be considered to withstand the temperature build-up in the conductive substrate (as an applied coating) during regular operations, *e.g.*, in the automotive parts. Here, polyether ether ketone (PEEK) and polyetherimide (PEI) have shown to be useful in terms of producing homogenous coatings and adequately allowing for thermal post-treatments performed. These post treatments are made to ensure uniform concealing and reformation of the insulating polymer characteristics, *i.e.*, post-coating deposition.^10–12^ Among the two, the PEI is the most interesting as it is the least expensive.

The EPD of PEI requires a quaternization reaction resulting in protonated PEI (qPEI) for the formation of a charged polymer emulsion suspension and subsequent effective electrophoretic deposition of the PEI onto the conductive substrate.^13,14^ The PEI's imide group undergoes a ring-opening reaction by the addition of 1-methylpiperazine, leading to the formation of an amide group in the PEI structure (Fig. 1a and S1†). The process is followed by acid protonation of lactic acid, and charge neutralization of the amine group, which in turn allow for the preparation of the polymer suspensions (emulsion) required for the EPD process (Fig. 1a). The inset in Fig. 1a shows the resulting polymer emulsion in the round bottom flask. After the EPD emulsion coating was carried out on the substrate, the coated substrate was treated at high temperature to allow the re-imidization of the PEI, producing the insulating coating (Fig. 1a). A challenge in this context is the possible H_2_ gas formation on the cathode electrode, from one of the half reactions in the electrolysis of water, and occasional formation of bubbles/voids in the formed PEI coatings.^15^ Therefore, alternating currents (AC) and pulsed direct currents (DC) were evaluated as process alternatives to improve the coating properties.^16^ However, to our best knowledge, enlightening comparisons between different voltages, current set-ups, surface substrate preparations, and charged polymer suspension formation have previously not been cross-examined, and compared, in terms of finding optimal conditions for generating useful EPD conditions and formation of uniform insulating coatings.

**Fig. 1:**
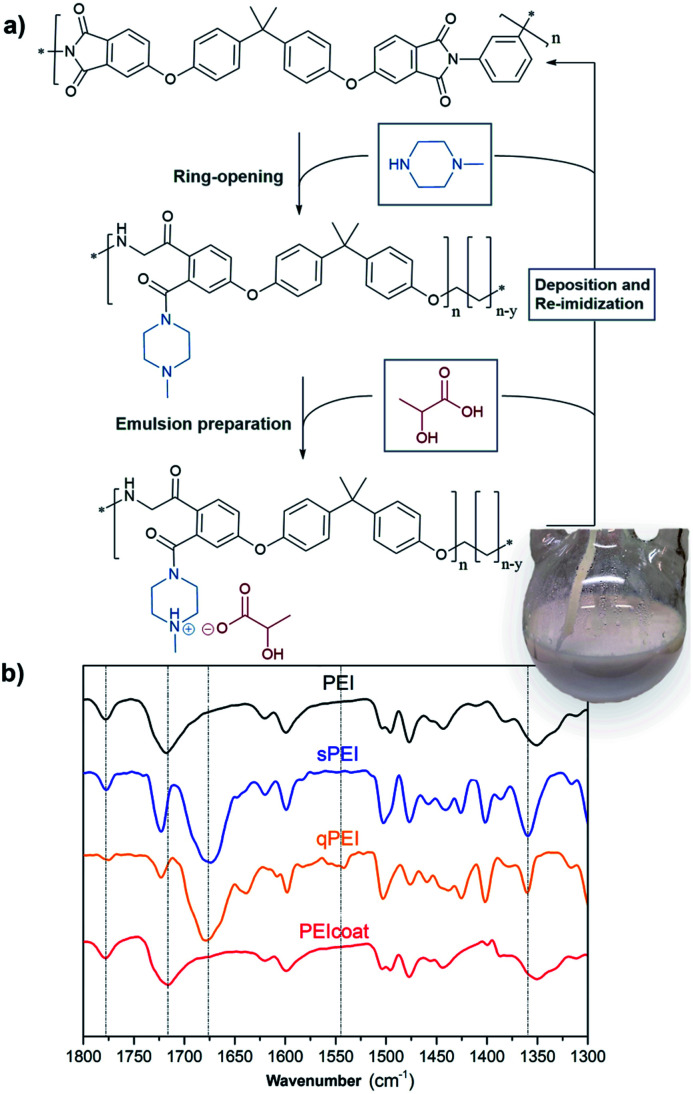
The electrophoretic deposition process from PEI to qPEI followed by re-imidization to PEI (a), and FTIR transmission spectra of the different samples (b). The coated PEI material was scrapped off prior to the FTIR analysis.

In this work, copper substrates with different surface treatments (polishing and sandblasting, Fig. S2†) and acidic cleaning (HCl and HNO_3_), were used for the EPD of qPEI under different potentials (2–20 V), see Fig. 2 and S3.† The selected potentials were obtained from screening useful conditions with associated electrical field strengths, as well as motivated from previous works.^17,18^ The qPEI suspension formation was evaluated in terms of lactic acid/water content and mixing temperature of the solution after the addition of water (Table S1†). The coated copper substrates with qPEI were always identically heat treated to re-imidize the qPEI forming the electrically insulating PEI layer (Fig. S4 and S5†). To prepare the qPEI, PEI (20 g) was dissolved in an NMP/acetophenone solution and 1-methylpiperazine was added. The solution was treated at 110 °C (2 h), which promoted the ring-opening of the imide ring in the PEI, from the solvated state (sPEI) to the fully protonated state (qPEI) (Fig. 1a and S1†). The changes in the FTIR spectra for the corresponding sPEI and qPEI is shown in Fig. 1b. The PEI stretching bands at 1780 and 1720 cm^−1^ (imide carbonyl of the five-member ring) and 1360 cm^−1^ (carbon–nitrogen vibration of imide five-member ring) were considerably reduced in the sPEI sample, and almost absent in the qPEI sample. Additionally, the strong band observed at 1670 cm^−1^ (–C=O vibration from amide) demonstrated the successful formation of qPEI (Fig. 1b).^14^ The band at 1360 cm^−1^ and the broad shoulder observed for the band at 1670 cm^−1^ was ascribed to the characteristics bands of acetophenone (Fig. S6†). The presence of the bands at 1780, 1720 and 1548 cm^−1^ confirmed the re-imidation of the qPEI forming PEI coating, see Fig. 1b.

**Fig. 2:**
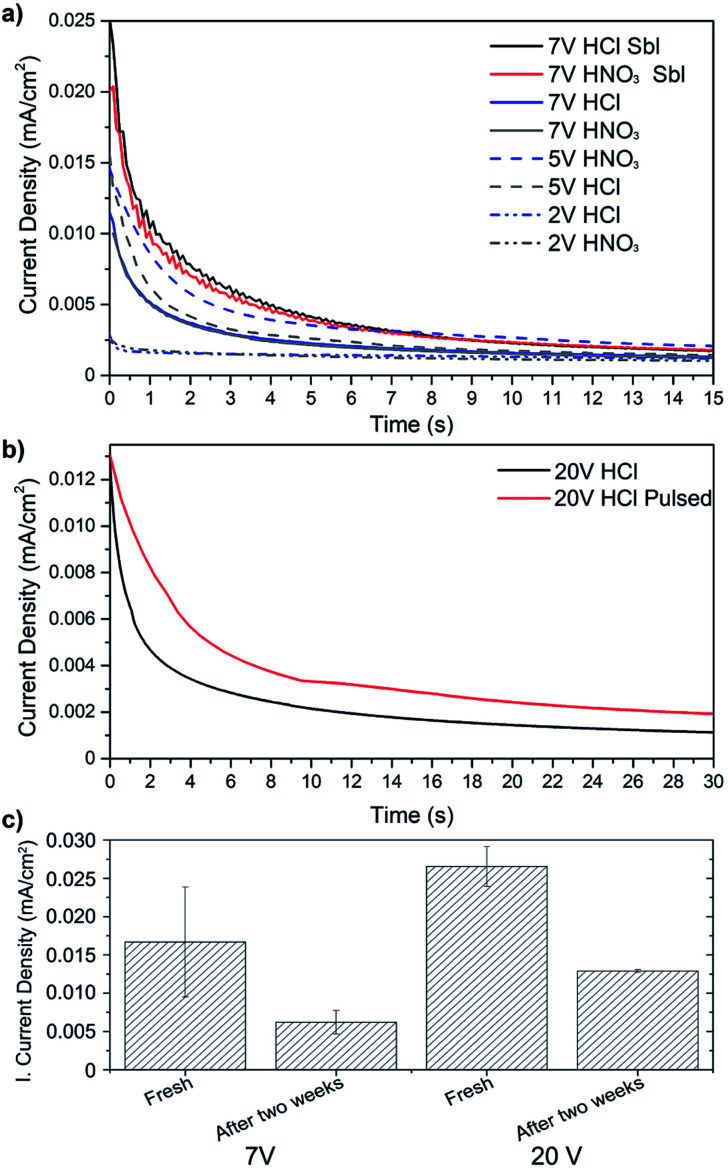
Current density decay on the copper substrate during the EPD process using different constant voltages and substrate pretreatments (a), compassion between constant and pulse voltage 20 V (b), and initial current density response when qPEI suspensions of different storage time were used (c). The result in “c” are the average of triplicates and the error bars the standard deviation.

The formation of a qPEI polymer suspension was strongly relying on the combined composition of lactic acid, acetophenone, and water, as well as the suspension temperature. The suspensions formed by 0.35 wt% lactic acid, 79.65 wt% acetophenone, and 20 wt% water, mixing temperature = 90 °C, (Suspension 1, Table S1†), resulted in solid lumps being formed at the bottom of the reactor (Fig. S7a†). Increasing the water content to 46 wt% on the expense of the combined amount of lactic acid and acetophenone (Suspension 2, Table S1†) resulted in a more homogenous suspension without apparent particle agglomeration at room temperature if rapid stirring was applied (Fig. S7b†). An increase of the lactic acid content to 1.3 wt% and water content to 67.5 wt% (Suspension 3, Table S1†) led to an unstable suspension that immediately phase separated, unless the suspension was stirred rapidly using mechanical stirring (Fig. S7c and d†). To stabilize the emulsion, the mixing temperature was decreased to 65 °C and the emulsion turned into a milky, highly viscous liquid (Suspension 4). At lower temperatures (23 °C or lower), a milky suspension with lower viscosity was formed (Suspension 5) that was stable for up to 48 h before any phase separation was observed (Fig. S8†). The pH of this optimized suspension was 4.6 and showed an electrical conductivity of 126 mS m^−1^. The addition of water to the qPEI was also found necessary to carry out as slow as possible to avoid excessive foam formation in the making of the optimized suspension, see further Fig. S9.†

The EPD process on the copper substrate using Suspensions 1 and 2 did not produce a coating layer on the substrate. Suspension 1 yielded a suspension with lumps formed whereas Suspension 2 appeared more homogenous (Table S1†) but no coating was formed, possibly due to limited ionic movement, which is a key factor/requirement for producing homogenous EPD coatings.^2^

Trials using Suspensions 3 and 4 for the EPD process revealed that an increased lactic acid content facilitated the formation of the coating, probably due to improved ionic movement, however none of these solutions produced homogenous coatings. Suspension 5 resulted in evenly covered copper substrates (Fig. S5†). The pH of the qPEI Suspension 3 was 8.6 ± 0.1 while for Suspension 5 was 4.6 ± 0.1. The difference in the pH for the aforementioned suspensions (both having the same lactic acid/water composition, Table S1†) could result from different zeta potential values, where very low zeta potential values have been previously showed to have a negative effect on the ionic stability/mobility in colloidal dispersions.^2^ Suspension 5 was therefore used for the further EPD of the copper substrates due to its low viscosity, low mixing temperature, and ability to form homogenous qPEI coatings on the substrate (Table S1 and Fig. S5†).

Fig. 2a shows the current density decay of the qPEI suspension during the EPD process for representative samples. The decrease in conductivity shown in Fig. 2a represents the coverage of the copper substrate (electrode) with the insulating qPEI layer. It should be noted that the decrease in current density was not ascribed to a depletion of qPEI in the solution/suspension. The same suspension could be used several times without an evident decrease in the initial current density. Fig. 2a shows that a sandblasted (Sbl) substrate coated at 7 V (constant voltage) resulted in double initial current density compared to the non-sandblasted substrate, independently of the pre-acid treatment used (7 V HCl Sbl – 7 V HNO_3_ Sbl *vs.* 7 V HCl – 7 V HNO_3_, respectively). The sandblasting process increased the surface area of the substrate, which was suggested to have favoured a higher exposure of the substrate in the qPEI suspension. Despite the increase in surface for the sandblasted samples, the current density equilibrium was always reached within less than 15 s, similarly to the non-sandblasted samples (Fig. 2a). The lower voltages in Fig. 2a were not further explored due to inefficient formation of the coatings and lack of electric field gradient in the suspension. A higher voltage of 20 V was instead considered but due to the unwanted half reaction, the pulsed voltage setting was explored. Although the use of 20 V pulsed voltage showed similar initial current density, the pulsed case presented a lower current density decay as compared to the constant 20 V settings, and the current density equilibrium was reached beyond 30 s for the pulsed sample (Fig. 2b). Fig. 2c shows the effect of keeping the qPEI suspension stored for extensive time (2 weeks) on the initial current density (prior to initiating the EPD process). The observed decrease in the initial current density for both 7 V and 20 V EPD treatment suggests that the number of charged particles in the suspensions decreased with longer storage times, which was a consequence of the qPEI emulsion aggregation. Accordingly, the initial current density obtained after storing the suspension for 2 weeks and using constant 20 V was similar to 7 V when used fresh (*ca.* 0.012 mA cm^−2^, Fig. 2c). The result show that for a production of qPEI substrate coating by EPD, the quality/thickness of the polymer coating is affected by the qPEI suspension storage time, but this may not be problematic as long as the voltage of the EPD is used to compensate for the reduced total charge of the emulsion.

Fig. 3a shows the result of an unsuccessful EPD coating when using the heterogeneous Suspension 3 (Table S1 and Fig. S7c, d†). The uneven coverage was revealed in the SEM micrographs, and the oxidation of the copper during the thermal treatment (re-imidization step) is visually observed in the image as a yellow/green coat (Fig. 3a). The sandblasting of the copper substrate in combination with coating from Suspension 5 (7 V HCl Sbl) is shown in Fig. 3b. The micrograph of the surface shows a homogenous and complete PEI coverage. No defects from the thermal treatment of the qPEI coated layer (to produce PEI by re-imidization, Fig. 1a) were observed (Fig. 3b). It is noteworthy that the PEI layer coated the rough sandblasted copper substrate perfectly around the periphery of the copper substrate, demonstrating the ability of the EPD process to evenly coat substrates of complex profiles.

**Fig. 3:**
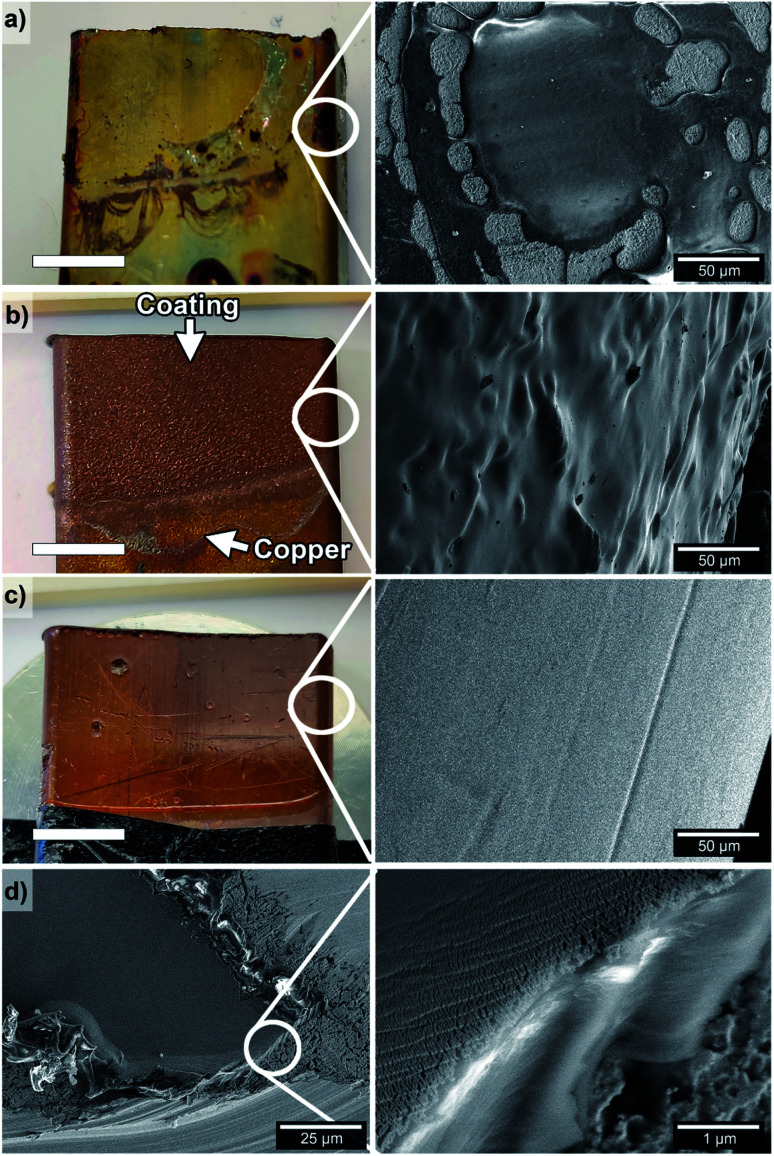
Copper substrate after the EPD process and thermal treatment (qPEI re-imidization) and SEM micrographs of the PEI coatings of samples coated (constant voltage) at 7 V HCl – Suspension 3 (a), 7 V HCl Sbl (b), 7 V HCl (c), and 7 V HCl coating layer after being scratched with a scalpel (d). The coatings in (b) and (c) firmly attached to the copper substrates.

Fig. 3c shows a copper substrate that was coated identically as Fig. 3b with Suspension 5, but in absence of sandblasting (7 V HCl). The microscopy revealed the smooth and evenly coated substrate obtained, with copper imperfections revealed through the coating (see photo Fig. 3c, left). A micrograph of the same coated 7 V HCl sample display how a scratch on the surface (with a scalpel after the coating procedure) appeared (Fig. 3d, left). The PEI coating layer had a thickness of about 3–4 μm on the copper surface and the coatings was revealed as firmly attached to the copper substrates, as seen from the encircled area shown with higher resolution imaging (Fig. 3d, right). Nano-sized cracks are observed on the coating close to the cut edge (Fig. 3d, right), which resulted from the plastic deformation of the PEI film coating. The results demonstrate the importance of using a homogenous suspension for obtaining well-distributed polymer and properly coated conductive substrates.

A typical defect observed in the EPD coated samples with the post-thermal treatment converting the qPEI to PEI (re-imidized) was the formation of bubbles in the coating film, see Fig. 4a. The effect was ascribed to volatiles formed at the surface of the copper as a consequence of hydrolysis occurring during the EPD process, leaving remains of gas (H_2_/O_2_) and/or hydroxyl groups that during the thermal treatment allowed condensation of water and resulted in poor consolidation of the coating.^15,19^ The formation of these bubbles was observed for all the samples after the drying of the coated substrate when using constant voltage for the EPD (60 °C oven for 17 h). On the contrary, the use of pulsed voltage (20 V) favoured the preparation of a smooth coatings on the substrate under the same drying conditions (60 °C oven for 17 h, Fig. 4b). Here, the presence of defects was limited to the edge of the sample (Fig. 4b). It is suggested that the pulsed deposition limited the hydrolysis reactions in the qPEI suspension due to the overall shorter time with applied potential, reducing the formation of hydrolysed fractions trapped between the coating and the substrate.^17,18,20^ However, more extensive drying of the coated copper substrate (60 °C oven for 44 h), also resulted in smooth coatings with no apparent defects, both for the constant 20 V and pulsed 20 V depositions (Fig. 4c and d). The longer drying times accordingly favoured the evaporation of the formed hydrolysed species and facilitated the release of entrapped volatiles. In industrial production, long drying times often represents excessive energy costs. The pulsed DC voltage is therefore suggested as a useful condition for generating smoothly coated substrates in short drying times. A stepwise build-up of the coating also enables otherwise entrapped volatiles to dissipate between each pulse when voltage is absent, resulting in a more homogenous morphology. The cross-section of the coated substrates (prepared *via* constant or pulsed voltage deposition) are shown in Fig. 4c and d, respectively. The EDS mapping of the coatings display the carbon base of the polymer, whereas the thickness could be established to around 4–6 μm.

**Fig. 4:**
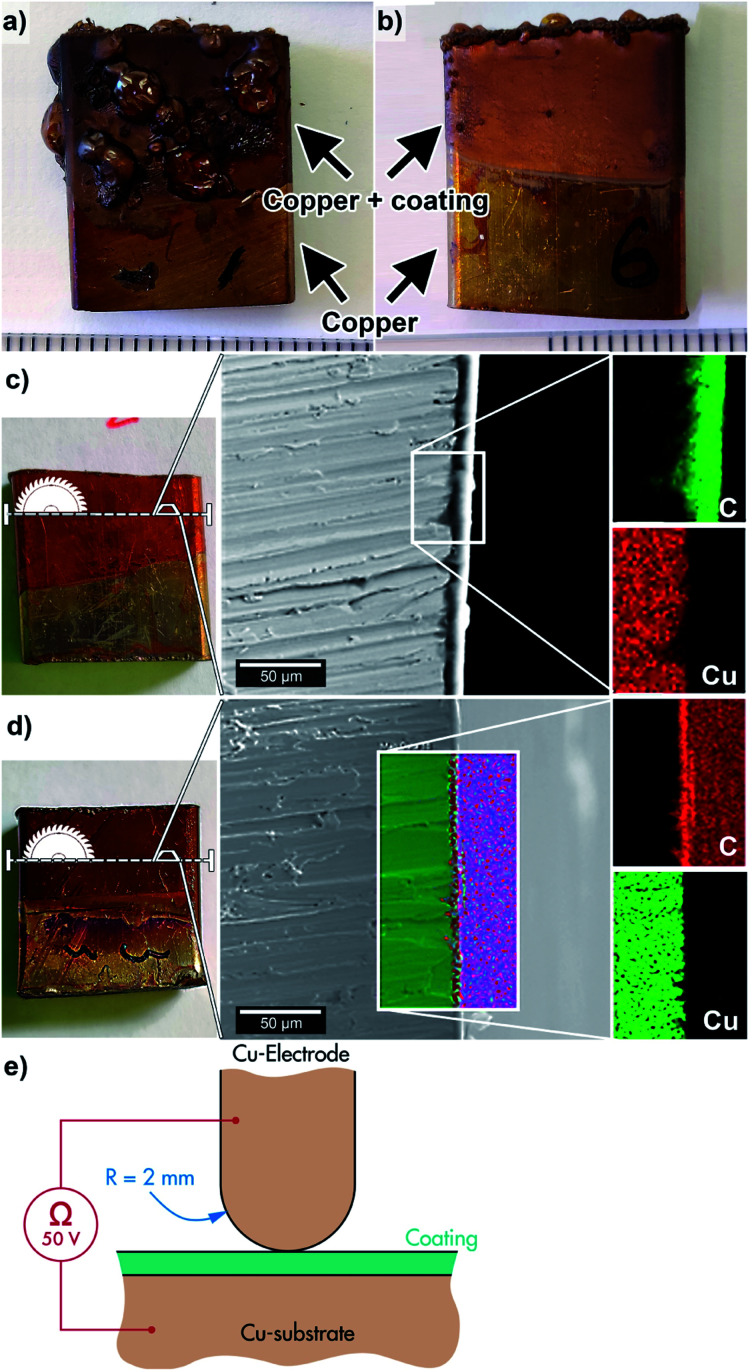
Shows the copper substrate coated without (a) and with (b) pulsed EPD conditions at 20 V, after for 17 hours of thermal post treatment at 250 °C. Images (c) and (d) show the same substrates after 44 hours 250 °C heat treatment, with accompanying SEM and EDS mapping insets; without pulsed 20 V deposition and with pulsed 20 V deposition, respectively. (e) Schematic representation of the electrical insulation resistance test setup. Note: it is not drawn to scale, as the coating is about 1/1000 the thickness compared to the size of electrode.

The DSC and TGA analysis performed on the removed PEI from the copper substrates showed that no significant difference in thermal properties resulted from carrying out the EPD process, *i.e.* when comparing the thermal characteristics of the virgin PEI before the quaternization reaction and the final PEI from the coating (Fig. S10a and b†). A slight decrease in the degradation temperature for the PEI retrieved from the coating layer as compared to the raw PEI (528 °C and 541 °C, respectively, Fig. S11 and S12†) was however observed. Although this could indicate some molecular changes in the PEI polymer on the coating and/or traces of low molecular weight fractions such as lactic acid (Fig. S12a and b†), these temperatures are well above the service temperature of electrically conductive copper cables (even considering heat build-up).

As an example of the insulating characteristics of the PEI coating, the electrical insulation of the coated and re-imidized samples, *i.e.* 7 V HCl sample (constant voltage), were evaluated using an insulation resistance test at a temperature of 20 °C (see Fig. 4e). The top electrode had a polished surface with a radius of 2 mm and the electrode was kept at a positive potential of 50 V with reference to the negative sample. For automotive applications, this voltage level may fit for VCA-systems (below 60 VDC).^21^ Considering that the coating thickness was on average *ca.* 5 μm, these conditions yielded a substantial electric stress of 10 kV mm^−1^ during the test. A Keithley 2450 source-measure-unit (SMU) was used to apply the voltage and to measure the corresponding current and resistance. Non-coated copper substrates showed a resistance of less than 0.1 ohm, essentially a short circuit (the SMU was current limited). The coated samples on the other hand showed an insulation resistance greater than 10^9^ ohm, illustrating the effectiveness of the EDP method and the re-imidization process in forming an insulating polymeric coating.

## Conclusions

Quaternization of PEI (polyetherimide), followed by the re-imidization of the qPEI into PEI, after electrophoretic deposition (EPD), is herein reported as a promising alternative technique (to dip coating) for developing insulating coatings on complex shapes and parts in the EV industry for automotive applications. The quaternized polyetherimide coatings are demonstrated as successfully applied onto copper substrates for a range of relatively low voltages (7–20 V), and the utilization of pulsed voltage was used to further promote coating smoothness. Among factors evaluated, firstly and most importantly, the temperature and agitation were of central importance for the preparation of a homogenous quarternized polyetherimide (q-PEI) emulsion suspension that was useful in the electrodeposition of the polymer onto copper substrates. The best conditions for achieving the homogenous suspension were here found to be at room temperature (*ca.* 23 °C), using vigorous stirring, which resulted in an electrical conductivity of 126 mS m^−1^ and a shelf-life of the suspension for about two days at room temperature. This q-PEI suspension was further demonstrated to allow for electrodeposition of 2–6 μm thick coatings at potentiostatic DC conditions of 2, 5, and 7 V, without pinholes and other irregularities. Pulsed DC was further introduced as a method for developing thicker coatings since it allowed for electrodeposition at higher voltages (>10 V). The pulsed deposition was at these potentials required because it limited the otherwise excessive hydrolysis occurring at the sample surface, not only causing cavities in the coatings but also alkaline etching of the copper substrate. The EPD technique is of interest to avoid the formation of coating pinholes/irregularities associated with dip or spray coating, which is of value while coating of complex parts within a variety of industries.

## Conflicts of interest

There are no conflicts to declare.

## Supplementary Material

RA-011-D1RA05448A-s001
