# Supplementary material for: Experimental review of PEI electrodeposition onto copper substrates for insulation of complex geometries
Source: RSC Adv. 2021 Oct 26;11(55):34599–604. doi: 10.1039/d1ra05448a (PMC9042725; doi:10.1039/d1ra05448a)
Supplement: RA-011-D1RA05448A-s001 [file RA-011-D1RA05448A-s001.pdf]

Electronic Supplementary Material (ESI) for RSC Advances.
This journal is © The Royal Society of Chemistry 2021

Supplementary Information

Experimental review of PEI electrodeposition onto copper substrates for insulation of complex geometries

J-C. Zirignon [a], A. J. Capezza [a*], X. Xiao [a], R. L. Andersson [a], M. Forslund [b],
P. Dinér [c] and R. T. Olsson[a*]

[a] KTH Royal Institute of Technology, Fibre and Polymer Technology Department, Teknikringen 56-58, SE-100 44, Stockholm, Sweden.

[b] Materials Technology department, YTME, Scania CV AB, Södertälje, Sweden.

[c] KTH Royal Institute of Technology, CBH, Department of Chemistry, Teknikringen 30, SE-100 44, Stockholm, Sweden.

[*]Correspondence: ajcv@kth.se +46 (0) 762301654; rols@kth.se +46 (0) 732701868.

Number of figures: 12

Number of pages: 12

1. Experimental

1.1. Materials

ULTEM™ RESIN 1000F3SP PEI Powder pellets was purchased from Sabic Basic Industries Corporation. Polyetherimide (PEI) pellets (MFI = 18 g/10 min), n-methylpyrrolidone (NMP, >99 %), lactic acid solution (LA, 85 %), acetophenone (>98 %), and 1-methyl piperazine (99 %) were all purchased from Sigma–Aldrich, Sweden. Hydrochloric acid solution (5M), nitric acid (ACS reagent 70 %), sodium hydroxide pellets (>98 %), sodium metasilicate, and sodium carbonate (99 %) were also purchased from Sigma-Aldrich. Copper rods (1.5 x 0.5 cm^2) were obtained from Scania CV AB, Sweden.

1.2 Quaternization reaction of PEI (q-PEI)

41.2 g of NMP (12.35 equivalents) and 5.14 g acetophenone (1.27 equivalents) were added to a flask and purged with argon for 20 min. Then, 20 g of PEI (1 equivalent) was added to two flasks and prepared separately. The flask was then heated to 90 °C (with a condensation unit reflux attached) under an inert argon atmosphere and constant stirring. The mixing continued until all the solid pellets were visually dissolved (between 14 to 36 h). A mixture of 15.4 g acetophenone (3.73 equivalents) and 4.5 g 1-methylpiperazine (1.4 equivalents) was prepared parallel and evenly added dropwise to the dissolution. The slow addition of acetophenone/1-methylpiperazine solution was complete after 2 hours. After that, the temperature of the flask was raised to 110 °C and kept for 2.5 h. The resulting q-PEI solution changed in color (from dark yellow to amber), as shown in Fig. S1.

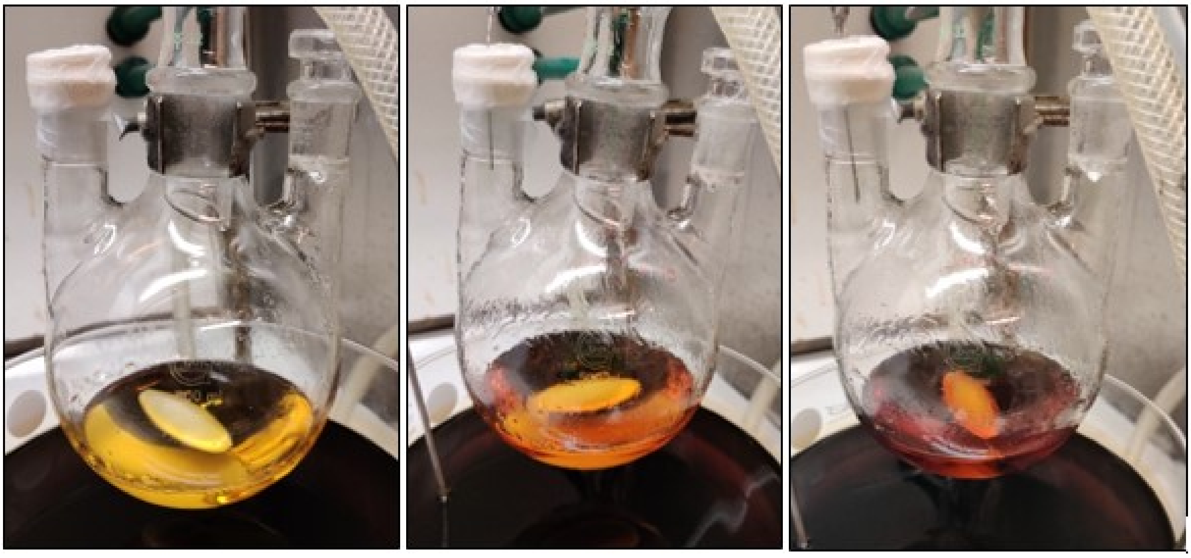

Fig. S1. Initial PEI solution (left), PEI solution after addition of 1-methyl-piperazine and 2 hours at 90°C (center), and the solution after the final heating state (2.5 hours at 110°C, right).

1.3 Preparation of the q-PEI suspensions for the coating

A mixture of 4.8 g 50 % lactic acid (1.41 equivalents) and 17.3 g acetophenone (4.26 equivalents) was prepared separately. The mixture was then added to the before described q-PEI solution (see section 1.2), and then 225 mL deionized water (371 equivalents) was added to the mixture under vigorous stirring (Suspension 3-5, Table S1). The addition of the water was performed both rapidly (during around 10 min) and slowly (during 50 min). To evaluate the effect of the mixing temperature for forming the q-PEI suspensions, the addition of the LA mixture to the q-PEI solution was carried out at room temperature (23 °C), 65°C, and 90°C. To evaluate the effect of the lactic acid (LA) concentration in the formation of the final suspensions, separate trials were prepared using:

i) Suspension 1: 0.35 wt% LA and 20 wt% water (corresponding to 0.37 g/0.12 equivalents LA, 1.5 g/0.37 equivalents acetophenone, and 19.9 g/ 32.1 equivalents water).

ii) Suspension 3-5: 1.3 wt% LA and 67.5 wt% water (corresponding to 4.8 g/1.41 equivalents LA, 17.3 g/4.26 equivalents acetophenone, and 225 g/371 equivalents water).

All the lactic acid dilutions used were prepared using deionized water and refluxed for 1 h at 120°C before their use.

Table S1. Experimental conditions for the preparation of the q-PEI suspensions.

Name	LA content (wt%)	Water content (wt%)	Mix. Temp. (°C)	Suspension characteristics
Suspension 1	0.35	20	90	Lumps formed
Suspension 2	0.24	46	90	Homogenous only under mechanical stirring
Suspension 3	1.3	67.5	90	Phase separation
Suspension 4	1.3	67.5	65	Highly viscous suspension
Suspension 5	1.3	67.5	23	Homogenous and stable even under mild agitation

1.4 Copper (substrate) preparation

The copper rods (1.5 x 0.5 cm^2) were cut using a saw to form 2 cm long pieces, and the edges/surfaces of the pieces were polished using abrasive sandpaper (3M Silicon sandpaper 600P and 1200P Wetordry). A non-polished and polished copper substrate is shown in Fig. S2 a and b. To evaluate the effect of the surface roughness on the coating quality, some of the pieces were sandblasted for 1 min using sand particles of 200-700 µm (Fig. S2c). All the polished copper pieces were washed with soap and water, dried, and then soaked in chloroform for ca. 30 min to dissolve grease traces. The samples were labeled with "Sbl" for those substrates that were sandblasted prior to the EPD.

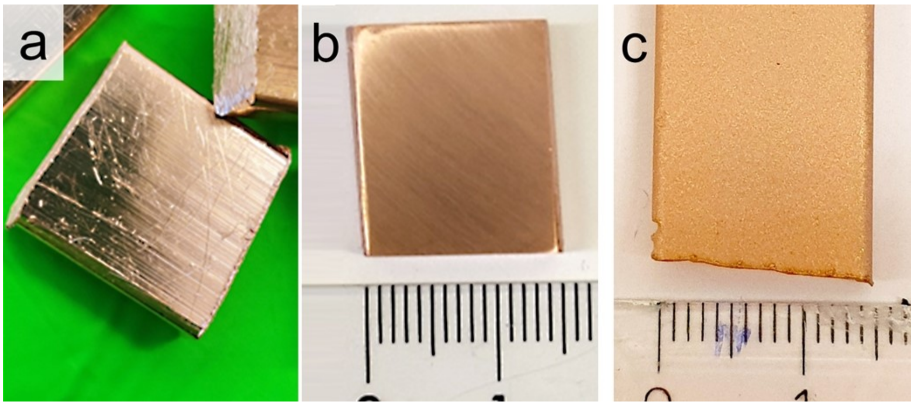

Fig. S2. Unpolished copper substrate (a), polished copper substrate (b), and sandblasted copper substrate (c).

The final copper substrate preparation before the electrodeposition consisted of sequentially immersing the samples in alkaline and acid solutions as follows: 15 min (alkaline), 5 min (acid), 5 min (alkaline), and 3 min (acid). The substrates were always rinsed with deionized water in between the different immersions. The alkaline solution consisted of a 40 g/l NaOH, 25 g/l sodium metasilicate, and 25 g/l sodium carbonate mixture, and it was heated to 90 °C before the immersion of the substrate. Two different acid solutions were used for rinsing the copper substrates, 5M HCl or 7 vol% HNO_3. The samples were with HCl or HNO_3 to specify the acid used for the rinsing.

1.5 Electrophoretic Deposition (EPD)

The electrophoretic deposition was investigated using a typical three-electrode cell setup, including a cleaned copper substrate as working electrode, a glass redrod electrode (1 M KNO_3, XR44 Radiometer Analytical) as the reference electrode, and circular platinum as the counter electrode. They were connected to a Keithly 2450 Sourcemeter. Approximately half of a circular platinum counter electrode (total area = 6.28 cm^2) was immersed in a glass beaker containing the suspensions prepared in section 1.3 (see, Fig. S3). The prepared copper substrate (described in section 1.4) was also immersed ca. 3.5 cm^2 in the q-PEI suspension. The distance between the platinum counter electrode and the copper substrate was always kept as 1 cm. The electrophoretic deposition was performed using constant, sweeping, or pulsed voltage (copper substrate electrode being negative in charge), and a mild stirring was employed throughout the entire process. An image of the whole experimental setup is shown in Fig. S3.

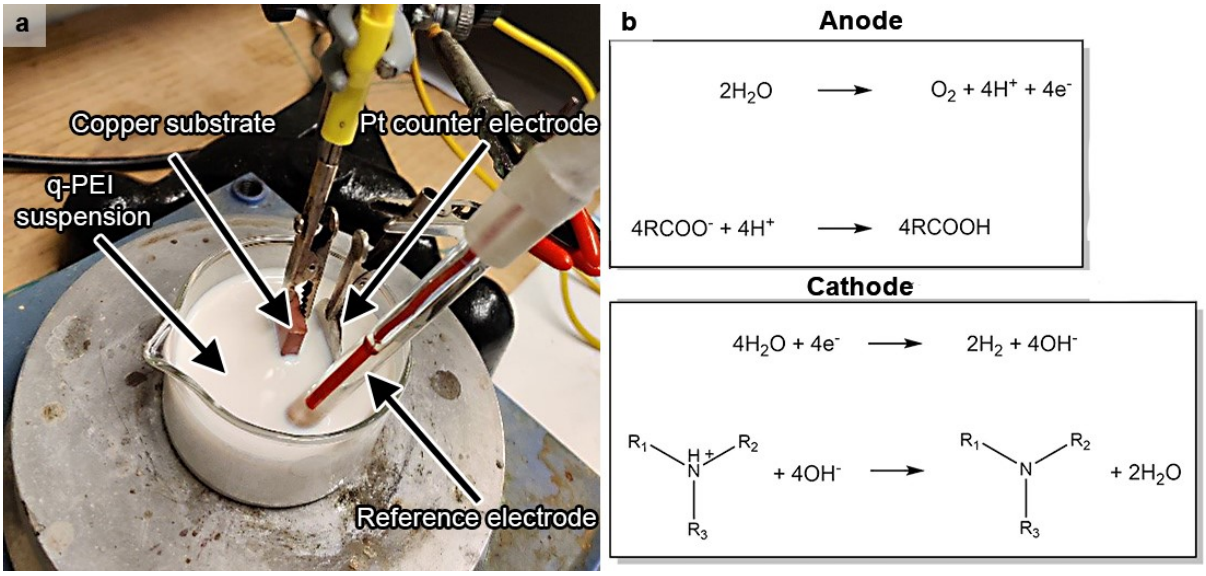

Fig. S3. Electrochemical cell setup (a) and possible reaction route following the consolidation of the charged polymer onto the copper surface during the electrodeposition (b). The reaction route was suggested by Qariouh et al. in previous works.[14]

For the constant voltage experiments, a voltage of 2, 5, 7, and 20V was applied for 10 s up to 10 min. The pulsed voltage trials consisted of pulses of 0 to 20V (at a frequency of 1 s) for 5 min. The voltage used is specified in the sample nomenclature, and "pulsed" is indicated in the sample labeling for those obtained at pulsed voltage.

1.6. Sample post-treatment

The prepared coated copper substrates were kept at 60 °C for 72 h for evaporating the remaining water and organic solvent. The samples were then placed in a glass cylinder, purged with argon (5 min), sealed, and placed in an oven at 250 °C for promoting re-imidization reaction of the q-PEI (Fig. S4). The samples were placed in the oven for 17, and 44 h. Fig. S5 shows a representative example of the copper substrate directly after the coating/drying at 60 °C and after the entire post-treatment process, Fig. S5 a and b, respectively.

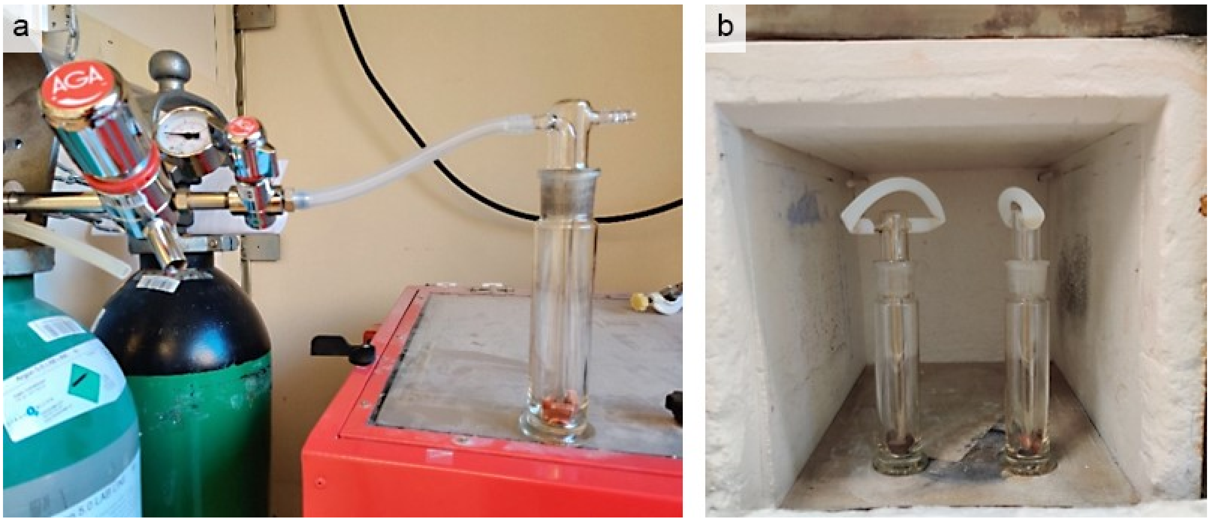

Fig. S4. Copper substrate sample during the argon purging (a) and the high-temperature treatment (250 °C, b).

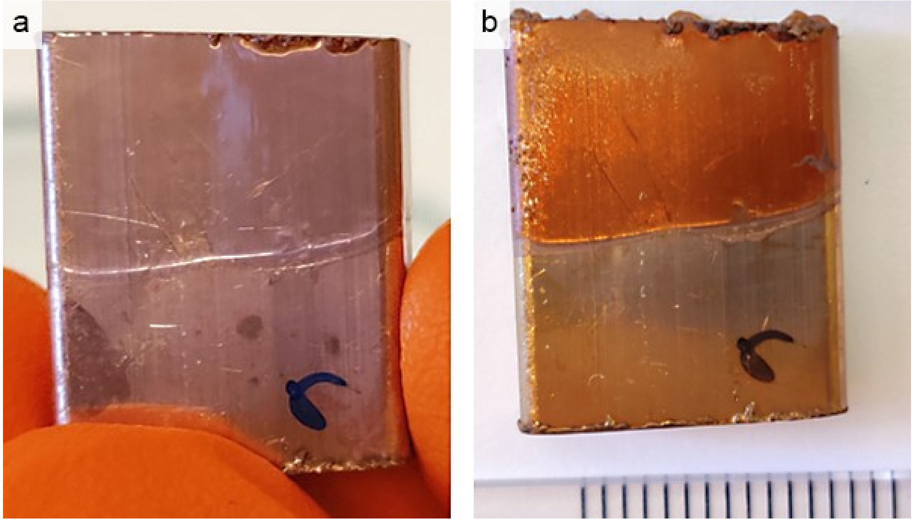

Fig. S5. A copper substrate (a) directly after coating with qPEI and (b) after the high-temperature post-treatment.

2. Analyses

2.1 Electrical conductivity and pH measurement

The pH and the electrical conductivity of the suspensions were monitored using a SevenCompact Duo s213 (Mettler Toledo, Switzerland).

2.2. Fourier Transformed Infrared Spectroscopy (FTIR)

The FTIR of the different samples and reagents was obtained using a Spectrum 100 (PerkinElmer, England) equipped with a single-reflection ATR unit (Graseby Specac Ltd, England). The spectra were obtained from 32 scans (resolution 4 cm^{-1}) from 600 cm^{-1} to 400 cm^{-1}. To obtain the spectrum of the coating layers, the coat was scraped off from the substrate surface using a metal spatula.

2.3 Scanning Electron Microscopy (SEM) and Energy Dispersive X-ray Spectroscopy (EDS)

The surface morphology images of the different samples were assessed using a S-4800 field emission scanning electron microscope (FE-SEM, Hitachi, Japan). All the images were taken using a voltage of 5 kV and a current of 10 µA. The samples were coated with a Pt/Pd alloy for 45 s (ca. 2 nm conductive layer). The cross-section images combined with the EDS analysis were obtained using a Zeiss Sigma VP scanning electron microscope equipped with an Energy Dispersive X-ray Spectroscopy sensor (Zeiss, Germany).

2.4 Thermal analyses

The thermal stability of the scrapped-off coatings from the substrates was measured using a TGA/SDTA851 (Mettler Toledo, Switzerland). Ca. 15 mg of the coatings was loaded into alumina crucibles, and the analysis was performed at a heating rate of 10 K/min until 700 °C using N_2 gas as the inert atmosphere (50 ml/min).

The thermal transition of the coatings was also assessed here using a DSC 820 instrument (Mettler Toledo, Switzerland). Ca. 10 mg of the coatings and the raw PEI powder were loaded into 100 µl aluminum pans, and the results are presented as an average of duplicates.

2.5. Electrical analysis

The electrical properties of the coatings during the q-PEI deposition were measured using a 2450 Keithley sourcemeter equipped with a three-electrode electrochemical cell. The herein prepared copper substrates (see section 1.2) served as the electrode, the platinum disc as the counter electrode, and a glass redrod electrode (1 M KNO_3, XR44 Radiometer Analytical) as reference. The voltage was sourced into the suspension under an open-circuit potential program, and the respective current response was measured.

The current density was calculated by dividing the obtained current by the coated area of each substrate.

3. Results and Discussion

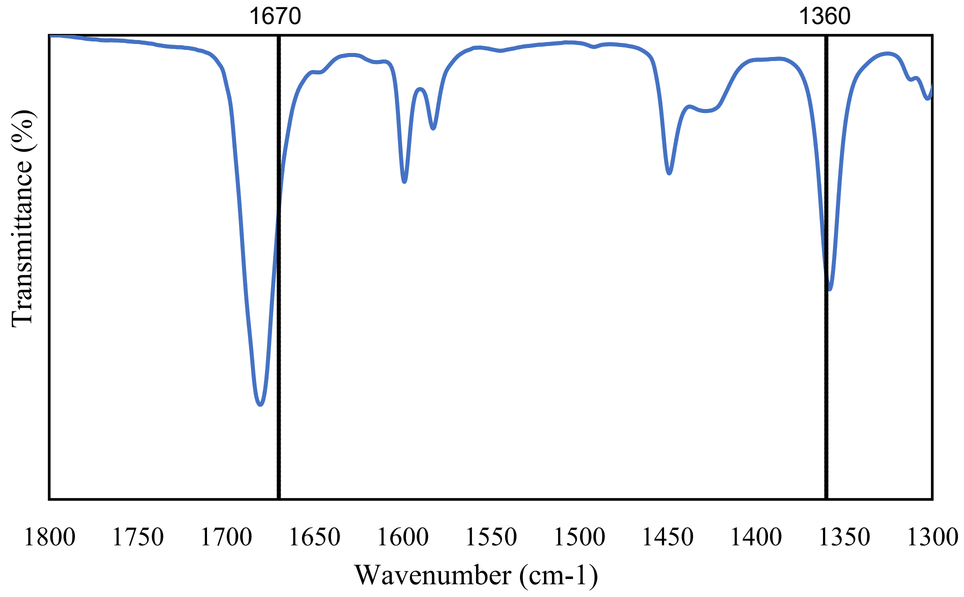

Fig. S6. FTIR spectrum of acetophenone.

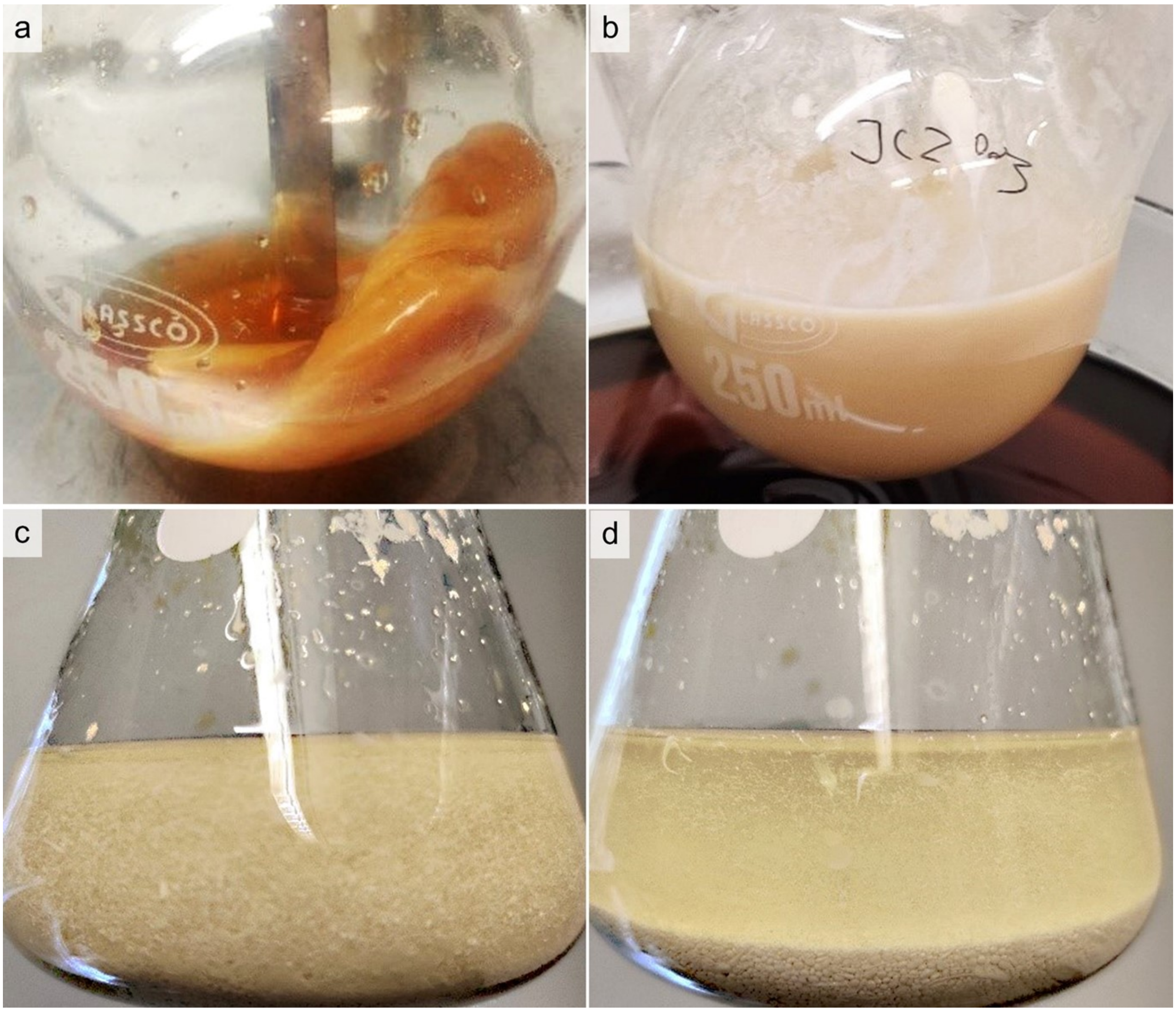

Fig. S7. 0.35 % lactic acid (LA) + 20% water qPEI Suspension 1 (a), 0.24 wt% LA + 46 wt% water qPEI Suspension 2 (b), and 1.3 wt% LA + 67.5 wt% water qPEI Suspension 3 directly after stopping the mechanical agitation and 10 s after (c and d, respectively). All these suspensions were prepared at 90 °C, according to Table S1.

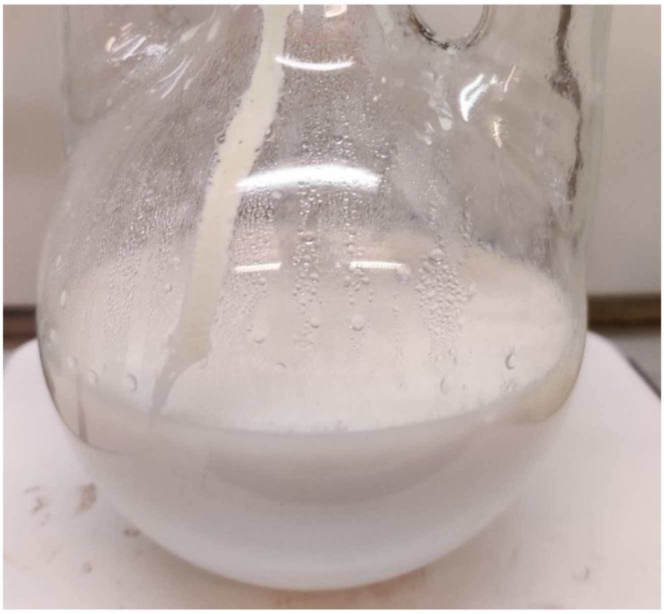

Fig. S8. 1.3 wt% LA + 67.5 wt% water qPEI suspension prepared at 23 °C (Suspension 5, Table S1).

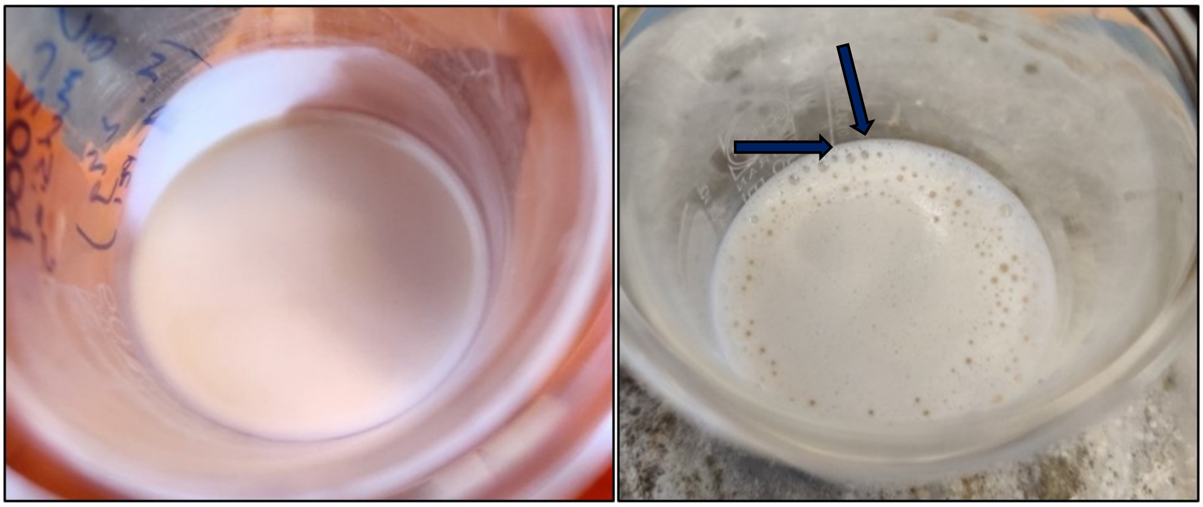

Fig. S9. The effect of “slow” and “rapid” addition of the water to the qPEI suspensions (described in section 1.3). The left image shows Suspension 5 after the slow water addition (total addition time was ca. 50 min), while the right image shows Suspension 5 after “rapid” water addition (addition time was ca. 15 min.). Arrows point out the foaming at the surface of the suspension in the right image.

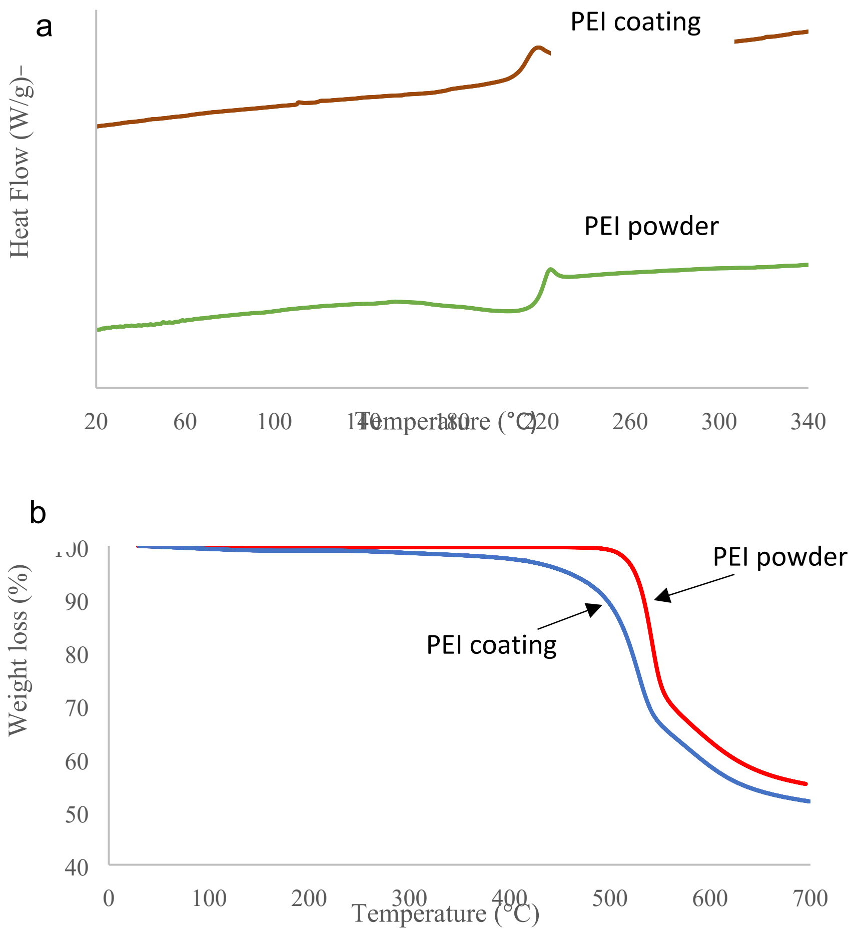

Fig. S10. DSC and TGA profiles the scratched PEI from the copper substrate (PEI coating) and the as-received PEI starting material (PEI powder), a and b, respectively.

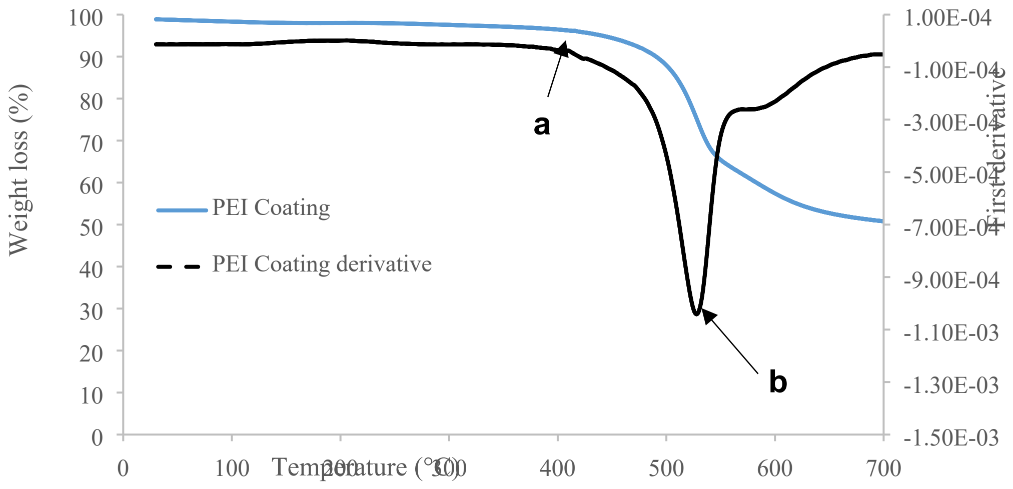

Fig. S11. TGA profile of the scratched PEI from the copper substrate (PEI coating, solid line) and the corresponding first derivative curve (dashed line). Significant thermal degradation (>loss than 2 wt.%) in the sample started at ca. 425 °C (a), and the maximum rate loss occurred at 528 °C (b).

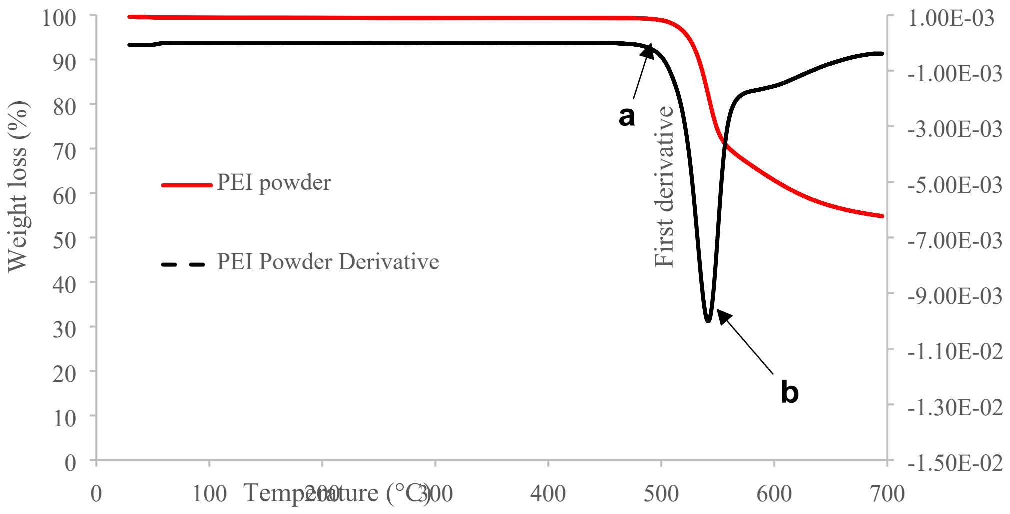

Fig. S12. TGA profile of the as-received PEI starting material (PEI powder, solid line) and the corresponding first derivative curve (dashed line). Significant thermal degradation (>loss than 2 wt.%) in the sample started at 495 °C (a), and the maximum rate loss occurred at 541 °C (b).
